# Brain Parenchymal Fraction: A Relatively Simple MRI Measure to Clinically Distinguish ALS Phenotypes

**DOI:** 10.1155/2015/693206

**Published:** 2015-12-13

**Authors:** Venkateswaran Rajagopalan, Erik P. Pioro

**Affiliations:** ^1^Department of Electrical and Electronics Engineering, Birla Institute of Technology and Science, Pilani, Hyderabad Campus, Hyderabad 500078, India; ^2^Department of Biomedical Engineering, ND2, Lerner Research Institute, Cleveland Clinic, Cleveland, OH 44195, USA; ^3^Neuromuscular Center and Department of Neurology, Neurological Institute, Cleveland Clinic, Cleveland, OH 44195, USA; ^4^Department of Neurosciences, Lerner Research Institute, Cleveland Clinic, Cleveland, OH 44195, USA

## Abstract

Even though neuroimaging and clinical studies indicate that amyotrophic lateral sclerosis (ALS) manifests with distinct clinical phenotypes, no objective test exists to assess upper motor degeneration in ALS. There is great interest in identifying biomarkers of ALS to allow earlier diagnosis and to recognize disease subtypes. Current quantitative neuroimaging techniques such as T2 relaxometry and diffusion tensor imaging are time-consuming to use in clinical settings due to extensive postprocessing requirements. Therefore, we aimed to study the potential role of brain parenchymal fraction (BPF) as a relatively simple quantitative measure for distinguishing ALS phenotypes. T1-weighted MR images of brain were obtained in 15 neurological controls and 88 ALS patients categorized into 4 distinct clinical phenotypes, upper motor neuron- (UMN-) predominant ALS patients with/without corticospinal tract (CST) hyperintensity on T2/PD-weighted images, classic ALS, and ALS with frontotemporal dementia (ALS-FTD). BPF was calculated using intracranial grey matter, white matter, and cerebrospinal fluid volumes obtained in control and ALS subgroups using SPM8 software. Only ALS-FTD patients had significant reduction in BPF when compared to controls and nondemented ALS patients. Correlation of clinical measures such as disease duration with BPF further supports the view that the BPF could be a potential biomarker for clinical diagnosis of ALS-FTD patients.

## 1. Introduction

Amyotrophic lateral sclerosis (ALS) is a progressive degeneration of motor neurons in brain and spinal cord of unknown cause [1]. Growing evidence from neuroimaging and clinical studies indicates that ALS manifests with distinct clinical phenotypes identified by extent of upper motor neuron (UMN) dysfunction [2], cognitive impairment (ALS patients with frontotemporal lobe dementia, ALS-FTD), and variable degrees of lower motor neuron dysfunction. According to the revised El Escorial criteria [3], the diagnosis of ALS is based on the presence of both UMN and LMN symptoms and signs. Whereas electromyography (EMG) is an objective test for LMN degeneration [4], no easily accessible equivalent exists to objectively identify UMN dysfunction in ALS, contributing to incorrect or delayed diagnoses [5]. There is great interest in identifying biomarkers of ALS to allow earlier diagnosis, recognize disease subtypes (which exist phenotypically), monitor disease progression, and assess the efficacy of therapeutic interventions.

Even though ALS patients have clinical evidence of both UMN and LMN dysfunction, a percentage of patients begin with UMN abnormalities before developing identifiable LMN signs. We have observed that some patients with predominantly UMN signs have bilateral corticospinal tract (CST) hyperintensities visible on conventional T2-, proton density-, and FLAIR-weighted image, while others with similar clinical features do not [2]. Why some patients with UMN-predominant ALS possess CST hyperintensities and others do not is unknown. Also, cognitive impairment in some patients with ALS affects predominantly frontotemporal areas to cause frontotemporal dementia (FTD) while prominent LMN dysfunction with UMN signs occurs in patients with classic ALS. Based on such observations, one can hypothesize different pathological mechanisms of ALS in UMN-predominant patients with or without CST hyperintensity, as well as those with combined UMN and LMN dysfunction or those with FTD.

Currently neuroimaging studies especially using MRI (because of versatile contrasts) to evaluate UMN dysfunction have provided better understanding of pathophysiological changes brought out by the ALS disease process. However, techniques such as T2 relaxometry, diffusion tensor imaging, and quantitative assessment of T1-weighted images using techniques such as voxel based morphometry (VBM) and cortical thickness analysis are time-consuming due to extensive postprocessing requirements. Therefore, these techniques have less widespread application clinically as opposed to research setting. On the other hand, measures such as brain parenchymal fraction (BPF) are not only quantitative but also simple and easy to calculate in clinical settings. Two previous studies in ALS [6, 7] found significant reduction in BPF of ALS patient brain; however, they did not categorize or classify ALS patients by their clinical phenotypes. Furthermore, they did not study the role of BPF as a potential clinical quantitative measure for distinguishing ALS phenotypes. We hypothesize that categorizing ALS patients by their clinical phenotype would reveal quantitative differences in BPF between such ALS subgroups and may identify the potential of BPF for distinguishing ALS phenotypes.

## 2. Methods

### 2.1. Demographics

MRI data obtained at 1.5 T during routine clinical neuroimaging were approved by the Cleveland Clinic Institutional Review Board for storage and analysis as deidentified images after patients (or their legal representative when they were cognitively impaired) provided verbal consent. The data were analyzed in the following patient groups: (1) neurologic disease controls (associated diagnoses indicated in Table 1); (2) UMN-predominant ALS patients with CST hyperintensity on T2/PD-weighted images (ALS-CST+) (this hyperintense signal is predominantly seen in posterior limb of the internal capsule (corresponding to corticospinal tract) and was identified by a blinded evaluator); (3) UMN-predominant ALS patients without CST hyperintensity identified on T2/PD-weighted images (ALS-CST–); (4) classic ALS (ALS-Cl); and (5) ALS with frontotemporal dementia (ALS-FTD). Representative demographics of the above patient populations are given in Table 2.

Patients who were identified by one of us (EPP) during clinical evaluation as having cognitive or behavioral impairment (e.g., disturbances of impulse control, executive function, and language) underwent formal neuropsychometric testing in most cases. Eighteen ALS patients met Neary criteria of FTD [8] after testing by an experienced neuropsychologist (*n* = 11) or bedside evaluation with MoCA (*n* = 7) and were included in the ALS-FTD subgroup.  Table 3 gives details of the domains affected in each of the ALS-FTD patients and their FTD subtype UMN-predominant ALS patients were defined as those with no lower motor neuron (LMN) signs or if present then these were restricted to only one neuraxial level (bulbar, cervical, thoracic, or lumbosacral) at the time of MRI. Classic ALS (ALS-Cl) had combined UMN and LMN features at one or more levels and did not display hyperintensity of CST. None of the ALS patients in the non-ALS-FTD subgroups had clinical evidence of FTD.

### 2.2. Clinical Data

Clinical measures of revised ALS functional rating scale (ALSFRS-R), disease duration (duration of symptoms prior to MRI), and disease progression rate were also measured and are given in Table 2. Disease progression rate was calculated by dividing the number of points ALSFRS-R score decreased from normal (i.e., 48) at the time of neuroimaging by symptom duration in months [9].

### 2.3. MR Image Acquisition

Structural high-resolution 3D T1-weighted MR images of head were obtained on a 1.5 T system (Siemens Symphony, Erlangen, Germany) using magnetization-prepared rapid gradient echo (MPRAGE) sequence. Imaging parameters were as follows: 160 slices, 1 mm thick, with 1.0 × 1.0 mm in-plane resolution; pulse sequence parameters were as follows: TR = 1970 ms; TE = 4.38 ms; number of averages = 1; and scan time = 6.45 minutes. T2- and PD-weighted images were also obtained using dual-echo FSE sequence to assess hyperintense signal changes along corticospinal tract in ALS patients. Imaging parameters include the following: 40 contiguous slices; slice thickness = 4 mm; in-plane resolution = 0.9 × 0.9 mm; pulse sequence parameters were as follows: repetition time (TR) = 3900 ms; echo time (TE) = 26 ms and 104 ms; echo train length or turbo factor = 7; and number of averages = 1; total scan time = 3.5 minutes. Although this dataset was used in our previous VBM studies [9], we did not study brain parenchymal fraction and so applied it to this study.

### 2.4. Brain Parenchymal Fraction Measurement

Whole brain intracranial GM, WM, and CSF volumes from T1-weighted images were obtained for control and the ALS subgroups using SPM8 software (http://www.fil.ion.ucl.ac.uk/spm). Since segmentation algorithms are automatic and are dependent on high GM/WM contrast, careful postsegmentation quality-checks were performed by an experienced neuroanatomist (EPP). BPF (in percentage) was obtained by taking ratio of brain parenchyma to the total brain intracranial volume [10] as given in

(1) Brain parenchymal fraction BPF=Volume of GM+WMVolume of GM+WM+CSF×100.

As seen from (1), change in BPF could result from one or both of the GM and WM parenchymal components. In order to further elucidate this, we studied separately the parenchymal fractions of GM and WM with respect to total intracranial brain, as given in (2). We have termed these as grey matter parenchymal fraction (GMPF) and white matter parenchymal fraction (WMPF):

(2) Grey matter parenchymal fraction GMPF=Volume of GMVolume of GM+WM+CSF×100,White matter parenchymal fractionWMPF=Volume of WMVolume of GM+WM+CSF×100.

### 2.5. Statistical Analysis

Clinical measures of revised ALS functional rating scale (ALSFRS-R), disease duration, and disease progression rate were compared between ALS subgroups using Kruskal-Wallis test with post hoc Mann-Whitney *U* test (using Bonferroni correction). BPF, GMPF, and WMPF measures were compared between control and ALS subgroups using ANCOVA by regressing out age, ALSFRS-R score, and disease duration. Multiple comparison corrections using Sidak test were performed with *P* < 0.05. Correlations between clinical measures (disease duration, ALSFRS-R, and disease progression rate) and BPF in ALS patients were performed using Spearman's rank correlation coefficient.

## 3. Results

**Figure 1:**
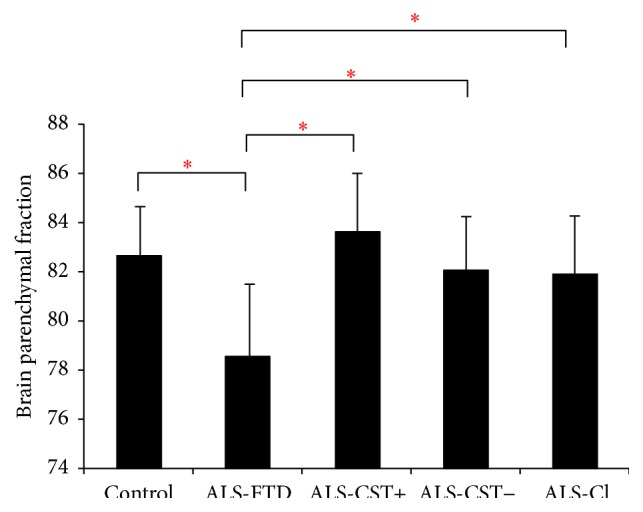
Brain parenchymal fraction values are significantly lower in patients with ALS-FTD compared to neurologic controls and other ALS subgroups. ^*∗*^ *P* < 0.05.

**Figure 2:**
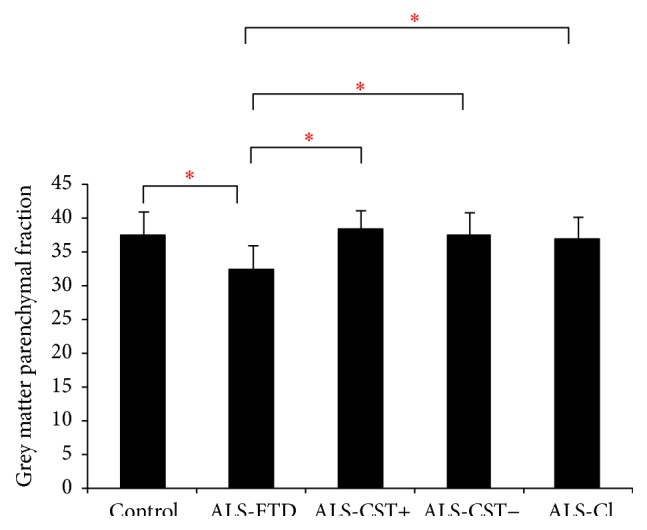
Grey matter parenchymal fraction values are significantly lower in patients with ALS-FTD compared to controls and other ALS subgroups. ^*∗*^ *P* < 0.05.

**Figure 3:**
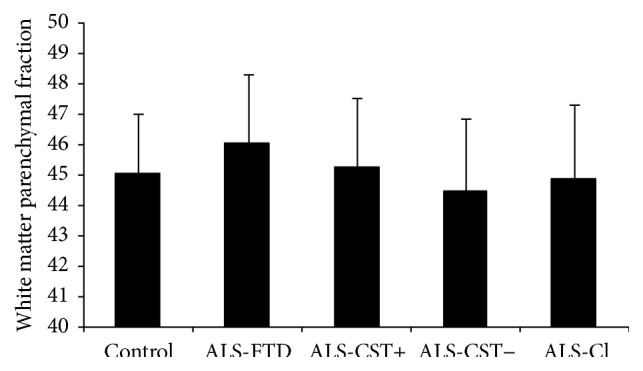
White matter brain parenchymal fraction values are not significantly different in any ALS patient subgroups compared to controls.

Significant (*P* < 0.05) reductions in BPF and GMPF were observed only between control and ALS-FTD groups as shown in Figures 1 and 2. Similar reductions in BPF and GMPF were significant in ALS-FTD patients when compared to other ALS subgroups (ALS-CST+, ALS-CST−, and ALS-Cl). However, WMPF showed no significant difference between controls and any of the ALS subgroups (Figure 3). Inability to discriminate the other patient groups from neurological controls may arise from some of these controls having a degree of cerebral atrophy from other neurodegenerative conditions (e.g., two with Parkinson disease). In order to evaluate this, statistical analysis was performed with the two parkinsonian patients excluded from the neurologic control group. However, we still failed to observe any significant differences between the control and other ALS subgroups/phenotypes, and the results remained the same whether Parkinson disease patients were excluded or not from the neurologic control group. Correlation between BPF and clinical measures revealed moderately significant positive correlation (*r* = 0.287, *P* = 0.005) between BPF and disease duration. No significant correlation was found between BPF and ALSFRS-R score (*r* = 0.143, *P* = 0.197), BPF and disease progression rate (*r* = 0.072, *P* = 0.521). No significant correlation was observed between WMPF and any of the clinical measures, that is, WMPF versus disease duration (*r* = −0.021, *P* = 0.843), WMPF versus ALSFRS-R score (*r* = 0.025, *P* = 0.816), and WMPF versus disease progression rate (*r* = 0.016, *P* = 0.886). No significant correlation was found between GMPF and disease duration (*r* = −0.197, *P* = 0.061), GMPF and ALSFRS-R score (*r* = 0.062, *P* = 0.55).

## 4. Discussion

The main findings of this study are as follows: (1) BPF was significantly reduced in ALS-FTD patients when compared to controls and nondemented ALS patients; (2) this reduction primarily arose from changes in the grey matter parenchymal fraction (GMPF) and not the white matter parenchymal fraction (WMPF); (3) BPF significantly correlated with clinical disease duration but not with ALSFRS-R score or with disease progression rate.

The present BPF results align with our previous findings of significant GM atrophy in only ALS-FTD patients as measured by VBM [9]. BPF reduction in ALS-FTD patients appears to result entirely from GMPF changes with no significant decrease in WMPF. The preferential reduction of GMPF in ALS-FTD patients also supports our previous hypothesis that GM atrophy results from a dying forward “neuronopathy” in such patients [9]. WMPF on the other hand is actually slightly* increased *in the ALS-FTD group (mean WMPF in neurological controls equals 45.06%, whereas in ALS-FTD patients it equals 46.05%), although not reaching statistical significance. This increase in WMPF could be due to gliosis that results in response to damage of WM axons and/or myelin. Similarly, our previous VBM analyses failed to reveal significant changes of subcortical WM in brain regions of ALS patients compared to control individuals [9]. In addition, we observed WM abnormalities in diffusion tensor imaging (DTI) metrics at rostral but not caudal levels of the corticospinal tract (CST) in nondemented ALS patients as revealed by fractional anisotropy (FA), axial diffusivity, and radial diffusivity values [11]. Lack of concordance between the DTI studies and WMPF findings in ALS patients may occur because (1) WMPF and WM VBM detect macroscopic changes whereas DTI identifies more microscopic changes resulting in earlier and more sensitive detection of pathology than do volumetric measures and (2) WMPF represents whole brain WM tracts while only the CST fiber tracts are included in our DTI findings. Taken together, abnormalities of CST DTI metrics (demonstrated previously) but not abnormalities of BPF, including GMPF measures (demonstrated in the present study), suggest that ALS-CST+, ALS-CST–, and ALS-Cl patients have less cortical pathology than do ALS-FTD patients.

In contrast to our findings, previous VBM studies in ALS found significantly reduced grey matter volume in nondemented ALS patients [6, 7], although at least some of these patients showed cognitive impairment clinically. Other possible reasons for these disparate results include the following: (i) combining various clinical phenotypes of ALS patients into the same group for analysis [6] rather than separating by distinct clinical phenotypes as in our study; (ii) studying patients with extensive disease burden and more advanced disease, for example, all with definite ALS [7] rather than ALS subgroups with relatively restricted LMN abnormalities (as in our study with patients average El Escorial score = 2, indicating laboratory-supported probable ALS); (iii) using neurologic disease controls rather than healthy controls, which may have introduced some degree of abnormality (e.g., atrophy) into our “control” group but alternatively represented a more appropriate (“real world”) comparison with ALS patients. Only healthy controls have been used in all other studies, making ours the first we know to have used neurologic disease controls.

Overall, ALSFRS-R values showed little difference among ALS subgroups suggesting that data were collected from patients with relatively similar degrees of functional impairment. However, significant differences were observed in disease duration among ALS subgroups. BPF and GMPF in ALS patients were significantly correlated with clinical markers of disease, including disease duration and disease progression rate. For example, positive correlation between disease duration and BPF suggests that shorter disease duration may be associated with worse disease and indicate both GM and WM damage.

Limitations of our study include the following: (1) lack of estimating the sensitivity and specificity of BPF, GMPF, and WMPF measures and (2) not evaluating changes in BPF, GMPF, and WMPF over time because of the cross-sectional nature of this study. Future longitudinal studies with larger sample sizes could confirm our findings.

MR imaging studies using techniques such as VBM, cortical thickness, and DTI showed significant GM and WM damage in ALS patients [6, 7, 12]. Although these techniques can certainly reveal abnormalities in* specific* brain regions as compared to BPF, which is a whole brain measure, they require extensive postprocessing of MR images, which is impractical in a clinical setting. Techniques such as VBM require robust registration to a template which in various pathological conditions (e.g., ventriculomegaly) may cause suboptimal normalization and segmentation leading to spurious results [13]. On the other hand Juengling and Kassubek [14] reported that BPF can not only be used for objective assessment of cerebral atrophy but can be included in MR reports of patients in routine diagnosis for neurodegenerative diseases. Along these lines we explored the use of BPF as a relatively quick and easy volumetric measure to distinguish ALS patients from controls as well as within ALS subgroups. Our results suggest that BPF, along with GMPF and WMPF, could serve as a potential MRI biomarker to distinguish ALS-FTD from other ALS subgroups in a clinical setting.

## 5. Conclusion

ALS patients with frontotemporal dementia have greatest reduction in brain parenchyma among ALS patients without dementia. Significant reduction in the GMPF and not the WMPF component of BPF suggests cortical atrophy and possibly a neuronopathy, in patients with ALS-FTD. Correlation of clinical disease duration with BPF further supports our suggestion that BPF and its individual components, GMPF and WMPF, may be useful MRI biomarkers for the clinical diagnosis of the ALS-FTD phenotype.

## Figures and Tables

**Table 1:** Clinical diagnoses of neurologic disease controls.

Subject	Clinical diagnosis
1	Severe fatigue, headache
2	Stiff person syndrome
3	Myasthenia gravis
4	Parkinson's disease
5	Depression, headache, and fibromyalgia-like syndrome
6	Fibromyalgia-like syndrome, headache
7	Painful sensory polyneuropathy
8	Insomnia, headache
9	Parkinson's disease
10	Cervical radiculopathy
11	Non-length-dependent small fiber sensory neuropathy
12	Headache, pain in lower leg
13	Small fiber neuropathy, headache
14	Large fiber neuropathy
15	Fibromyalgia-like syndrome, headache

**Table 2:** Demographics and clinical measures of neurologic disease controls and ALS patients.

Clinical measure/ALS subgroups	Neurologic disease controls	ALS-CST+	ALS-CST−	ALS-Cl	ALS-FTD
*n*	15	21	26	23	18

Age (years) (mean ± SD)	57.1 ± 19.2	52.3 ± 11.02	60.1 ± 11.8	58.5 ± 12.6	66.4 ± 9.2

Age range (years)	28–95	32–75	32–76	39–84	52–87

Gender	10 men, 5 women	14 men, 7 women	13 men, 13 women	13 men, 10 women	5 men, 13 women

Duration of symptom prior to MRI (months) (mean ± SD)		9.6 ± 5.5	36.4 ± 44.2	29.1 ± 27.3	37.5 ± 25.2

ALSFRS-R score (*N* = 48) (mean ± SD)		34.6 ± 7.8	34.1 ± 8.1	37.2 ± 8.5	30.7 ± 7.1

Disease progression rate (mean ± SD)		1.38 ± 1.64	0.46 ± 0.43	0.68 ± 0.77	0.59 ± 0.33

**Table 3:** Clinical characteristics of ALS patients with FTD.

Patient	Gender	Age (yr)	Site of onset	Features of FTD	
				Extent	Domain affected
					(at time of MRI)
1	F	67	Speech	Mild	bv., ex., l.
2	F	75	UE	Mild	ex., l.
3	F	60	Speech	Mod.	bv., ex.
4	F	58	Speech	Mod.	bv., l.
5	F	60	Cognitive	Mod.	bv.
6	M	63	Speech	Severe	bv., l. (PNFA)
7	M	75	Cognitive	Severe	bv., mem.
8	F	53	Speech	Severe	bv., l.
9	F	52	Speech	Severe	g.
10	M	59	Cognitive	Severe	g.
11	M	87	UE, LE	Severe	g.
12	F	69	Cognitive	Severe	bv., l.
13	F	68	Cognitive	Severe	bv., ex.
14	F	67	LE	Severe	ex., l. (sem.), mem.
15	F	65	Cognitive	Severe	bv., l.
16	F	63	LE	Severe	bv., ex., l.
17	F	77	Speech	Severe	bv., ex., l.
18	M	78	LE	Severe	g.

bv. = behavior; Cog. = cognitive; ex. = executive; g. = global (bv. + ex. + l. + mem. present); l. = language; LE = lower extremity; mem. = memory; PNFA = progressive nonfluent aphasia; sem. = semantic; and UE = upper extremity.
